# Investigation of customized total knee implant with articular cartilage under loading conditions using finite element analysis

**DOI:** 10.1371/journal.pone.0311210

**Published:** 2025-02-03

**Authors:** Shady A. Alshewaier, Mohamed Yacin Sikkandar, Ali Ahmed Ali Almakrami, S. Sabarunisha Begum, Ahmad Alassaf, Ibrahim AlMohimeed, S. Meenatchi Sundaram, Dheeraj Poojary, Natteri M. Sudharsan, Eddie Y. K. Ng

**Affiliations:** 1 Department of Physical Therapy‬, College of Applied Medical Sciences‬, Majmaah University, Al Majmaah, Saudi Arabia; 2 Department of Medical Equipment Technology, College of Applied Medical Sciences, Majmaah University, Al Majmaah, Saudi Arabia; 3 Department of Biotechnology, P.S.R. Engineering College, Sivakasi, India; 4 Department of Instrumentation and Control Engineering, Manipal Institute of Technology, Manipal, India; 5 Machine Design, NMAM Institute of Technology Nitte, Udupi, India; 6 Department of Mechanical Engineering, Rajalakshmi Engineering College, Chennai, India; 7 School of Mechanical & Aerospace Engineering, College of Engineering, Nanyang Technological University, Singapore, Singapore; University of South Carolina, UNITED STATES OF AMERICA

## Abstract

Knee osteoarthritis (KOA) is a degenerative joint disease predominantly affecting the elderly and is often managed through knee replacement surgeries. West Asians, who frequently engage in activities involving bending and kneeling during their prayers, tend to exhibit distinct bone anatomy compared to the Caucasian population. This research posits that patient-specific, customized knee implants with articular cartilages may lead to reduced post-surgical discomfort and a better implant fit compared to conventional standard implants. This study presents a novel concept and approach for the development of a customized total knee implant with articular cartilages, specifically tailored to simulate loading conditions using finite element analysis (FEA) for the Saudi Arabian population. The research analyses patient-specific customized knee implants with articular cartilages under both pre- and post-implant conditions using a finite element model (FEM). Computed tomography (CT) images of patients were utilized to create a solid model, which was then analysed under various constraints and conditions. The meshing process employed tetrahedral elements, converging with 76,197 nodes and 43,009 elements. The analysis was conducted under different body weights, specifically 75–80 kg and 100–105 kg. Two sets of force and moment were applied: the first with a force of 1000 N and a moment of 1.5 N-m, and the second with a force of 750 N and a moment of 0.8 N-m. The results indicated a 5% reduction in stress with implants designed for a 100 kg body weight, along with a significant reduction in ligament strain when compared to conventional knee joint stresses. This study offers a promising pathway toward reducing post-surgical discomfort. The proposed innovative solution has the potential to revolutionize total knee implant technology, offering enhanced functionality and improved patient outcomes for the Saudi Arabian population.

## 1. Introduction

The knee joint is among the most vulnerable and susceptible joints in the human body [1]. Knee osteoarthritis (KOA) is a degenerative joint disease predominantly affecting the elderly, arising from biomechanical stresses that impact the articular cartilage and subchondral bone of the knee [2]. KOA affects 7% of the global population, with the number of cases worldwide increasing by 48% between 1990 and 2019 [3]. In 2019, KOA ranked as the 15th leading cause of years lived with disability (YLDs) globally, accounting for 2% of total global YLDs.

The preferred treatment for this degenerative joint disease is knee implantation, which ranges from partial to total knee replacement (TKR) [4]. Key drivers of TKR demand include obesity, an aging population, and increasing life expectancy [5]. However, traditional knee implants are typically available in a limited range of standard sizes and shapes. Most total knee arthroplasty (TKA) implants currently on the market are specifically designed to fit the knee anatomy typical of Western populations [6]. Our review indicates that implant manufacturers offer various designs that preserve some ligaments while replacing others, and no single implant can be definitively regarded as superior to the rest. As each bone architecture is unique in terms of surface profile, the off-the-shelf implants must be fixed by tailoring the bone by grinding and cutting. This is traumatic and very painful with longer recovery. The current proposal to scan the bones and customize the implant will substantially decrease the extent of bone alteration required. Furthermore, bone degradation is influenced by an individual’s gait and lifestyle. By personalizing the implant, much like custom orthopedic footwear, patients can achieve a significant enhancement in their quality of life. Research has consistently shown that Caucasian knees tend to have a relatively low posterior tibial slope, typically ranging from 3° to 7° [7, 8]. This lower slope is aligned with lifestyle patterns that involve less frequent deep knee flexion activities, such as squatting or kneeling. Consequently, the knee anatomy in Caucasians is more adapted to movements involving moderate flexion, such as walking or running, rather than deep bending [9]. In contrast, Saudi and Asian populations exhibit a higher posterior tibial plateau slope, often ranging from 7° to 12° or even higher [10, 11]. This steeper slope is primarily attributed to cultural practices that involve regular squatting, kneeling, and deep flexion of the knee, such as sitting on the floor, prayer postures, and other daily activities. These practices place significant biomechanical demands on the knee joint, leading to an adaptive increase in the posterior slope to better distribute loads during deep flexion [12, 13]. The differences in posterior tibial slope between these populations have significant implications for knee function and surgical interventions. A steeper posterior slope in Saudi and Asian knees allows for greater flexion and helps accommodate the stresses associated with activities that involve deep knee bending. However, it also places different biomechanical demands on the knee, potentially affecting joint stability and the risk of injury. So, there is a need for customized implants for region-specific patients as the anatomy is different and implants need to be based on ethnicity and lifestyle. Moreover, after a few years of TKR surgery with standard implants, some patients may need to replace their previously implanted knee for various reasons such as misalignment and improper fit of the implantation component [14]. After a few years of TKR surgery, some patients may need to replace their previously implanted knee for various reasons such as misalignment and improper fit of the implantation component [14]. In addition, the occurrence of rotational errors within the knee components, especially the tibia, during TKR surgery contributed to the experience of discomfort and reduced functionality in the knee joint afterward. It is crucial to comprehend the motion of the knee joint during various activities to achieve a prosthetic knee that closely mimics the normal joint function. This understanding will enhance TKR procedures aimed at restoring the patient’s knee joint geometry to its anatomically correct state. Also, a proper evaluation of the damages and limitations of function after surgeries would help improve the design of a rehabilitation course by specialists. However, with advances in imaging technology and computer-aided design, TKR would be possible to create patient-specific knee implants that closely match the individual’s anatomy. The customization knee implant allows for a better fit and potentially improves the procedure outcomes for the patient.

Finite element analysis (FEA) is a computational technique used to analyze the behaviour of complex structures and systems under various conditions. FEA has enabled researchers to study knee joint dynamics [15, 16], and simulate various loading conditions that the knee joint experiences during daily activities such as walking, running, or climbing stairs [17, 18]. By subjecting the customized implants with articular cartilages to these loading conditions, the performance assessment, including stresses and strains on different components of the implant, as well as the surrounding tissues would be possible [18]. Such FEA analysis can aid in optimizing implant design, material selection, and surgical techniques to improve longevity and functionality. Yash Shah et al. (2015) utilized a prototype as a reference to model and simulate the knee under different stress and strain conditions. Their objective was to analyze the fatigue life of the joint and assembly, aiming to determine the most effective treatment for knee disorders among the recommended options for patients [19]. Oshkour et al. [2015] worked on a parametric analysis of radial functionally graded femoral prostheses across various geometries using FEA [20]. The findings demonstrated that the strain energy density, interface stresses, and stresses within the implanted femur component are influenced by the gradient index and the geometric characteristics of the prostheses. Martin Kubicek et al. (2009) focussed on the stress-strain analysis of the normal tibia-femoral joint at its normal position (extension). Through their analysis, the researchers successfully derived the contact pressure between the cartilage of the femur and tibia, as well as between the femoral cartilage and meniscus. They utilized CT images to create a model that enabled them to evaluate the contact pressure among the knee, cartilage, and meniscus. The solution to this problem was attained by employing ANSYS software [21]. In their research, Ho-Jung Cho and Dai-Soon Kwak (2020) conducted a study on the major knee ligaments, including the anterior cruciate ligament (ACL), posterior cruciate ligament (PCL), medial collateral ligament (MCL), and lateral collateral ligament (LCL). They examined the mechanical properties of the ACL, MCL, and LCL, specifically focusing on the relative differences between these ligaments [22]. Their findings indicated that gender differences were noticeable primarily in terms of the ultimate load borne by all ligaments. The study aimed to emphasize the significance of ligament stiffness in achieving optimal outcomes. Iliana Loi et al. (2021) presented a semi-automatic framework to create subject-specific TKR finite element (FE) models to analyze locomotion patterns and evaluate knee dynamics [23]. The primary objective of their study was to explore the potential for customization of total knee replacement (TKR) surgeries using simulations. The aim was to minimize post-operative complications. The study successfully demonstrated that the alignment of the femoral component relative to the knee bones has a significant impact on load distribution at the tibiofemoral joint. However, it is important to note that the study lacked comprehensive information regarding the parameters required for structural analysis. There are few research works carried out on morphological analysis using FE models [24–26] and limited literature available to address the need for complete structural analysis of knee joints with customized implants under loading conditions [27–29]. A simplified FEA of customized knee implants during flexion-extension movement was developed by Shady et al. [30]. However, the study was a preliminary analysis with fixed numbers of mesh numbers and there was no consideration of varied force and moment and the articular cartilages. Much of the existing literature on customized knee implant analysis was carried out on the western population and limited studies were carried out on the Asian population. To our knowledge, none of the study conducted on the Saudi Arabian population. This constraint necessitated us to carry out a detailed analysis to overcome the limitations in the existing studies. The previous study [30] has been now expanded by incorporating the analysis of articular cartilages under different loading conditions. This new aspect provides deeper insights into the biomechanical behaviour of customized total knee implants, which was not covered in the earlier study. In this study, a realistic and detailed structural analysis by applying load and moment with articular cartilages has been studied to address the limitations of conventional knee implants. In the current work, the implant was reverse engineered to suit the anatomy based on the work of Lin et al. [31], and hence, here the effort was to show the advantage of developing customized implants for individual subjects. By conducting thorough research and employing advanced computational modelling techniques, the authors have devised an innovative methodology that revolutionizes the design process. This methodology integrates patient-specific data and considers crucial biomechanical factors, resulting in a significant advancement in the field.

### 1.1. Hypothesis of the research

This research focuses on the analysis of patient-specific customized knee implants for the Saudi Arabian population, utilizing FEA under realistic loading conditions. Realistic loading is essential for evaluating the performance of knee implants in scenarios that closely mimic real-life activities. By simulating the forces and loading patterns experienced by the knee joint during activities such as walking, running, and climbing stairs, researchers can assess the implant’s functionality in dynamic situations. This evaluation is critical for determining the implant’s durability, stability, and long-term success.

The research addresses the issue of improper implant fit, which can lead to discomfort and uneven load distribution. An ill-fitting knee implant may result in joint instability, potentially undermining the knee’s support during movement and compromising joint stability. Such instability can restrict a patient’s ability to perform daily activities and might necessitate additional interventions. Furthermore, an inadequately fitting knee implant could accelerate wear, leading to earlier-than-expected revision surgery. By examining patient-specific implants, this study aims to alleviate post-surgical discomfort and enhance implant fit, thereby improving clinical outcomes. The significance of this research lies in advancing the management of knee osteoarthritis through the customization of total knee implant designs, refining treatment strategies, and increasing patient satisfaction. The research hypothesis posits that patient-specific customized knee implants with articular cartilages will result in reduced post-surgical discomfort and superior implant fit compared to conventional standard implants.

The study seeks to analyze the biomechanical effects of customized knee implant kinematics under varying body weights in both pre- and post-surgical conditions. The objective is to develop a realistic customization approach using FEA, which includes the biomechanical assessment of articular cartilage under loading conditions. The proposed approach aims to facilitate the design, evaluation, and optimization of total knee implants.

## 2. Materials and methods

### 2.1 Data collection

In this study, computed tomography (CT) images were obtained from 15 osteoarthritis subjects at King Fahad Medical City (KFMC), Riyadh, Saudi Arabia in the age group (60 and 80 years including both Males and Females) were collected during the year 03 March 2022 to 02 March 2023 and the data were authorized to use for research purposes only. The ethics committee waived the need for informed consent. Authors do not have access to information that could identify individual participants during or after data collection. This study has received approval from the KFMC Institute Review Board (IRB) under IRB Log Number: 22-032E.

**Fig 1:**
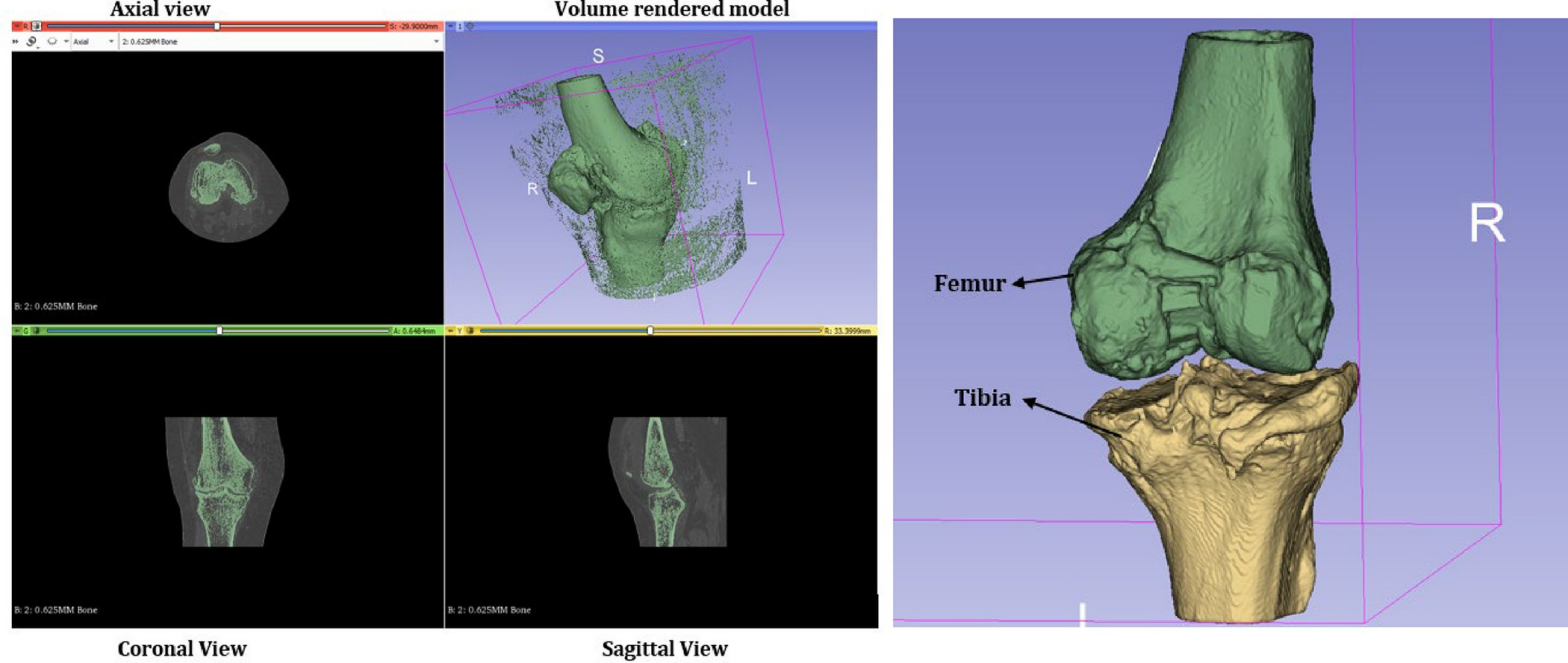
a) Segmentation process of DICOM file b) Volume rendered 3D model.

The Study began with the initial CT scans of patients in Digital Imaging and Communications in Medicine (DICOM) format image which consists of image slices at axial, coronal, and sagittal orientations of the knee joint. The DICOM image file contained a total of 531 slices of 0.625 mm Bone with dimensions 512 mm X 512 mm X 531mm and spacing of 0.324 mm X 0.324 mm X 0.31 mm for volume rendering. Fig 1 shows the segmentation process of the DICOM image file resulting in a volume-rendered 3D model. For the 3D model, the selected threshold range was between 117.35 (minimum value) and 2333.60 (maximum value). The noises and unwanted traces in the model were removed afterward through the post-processing tool in the 3D slicer software. The images presented here are from the CT images of a male subject of 68 years old.

### 2.2 Surface model preparation

**Fig 2:**
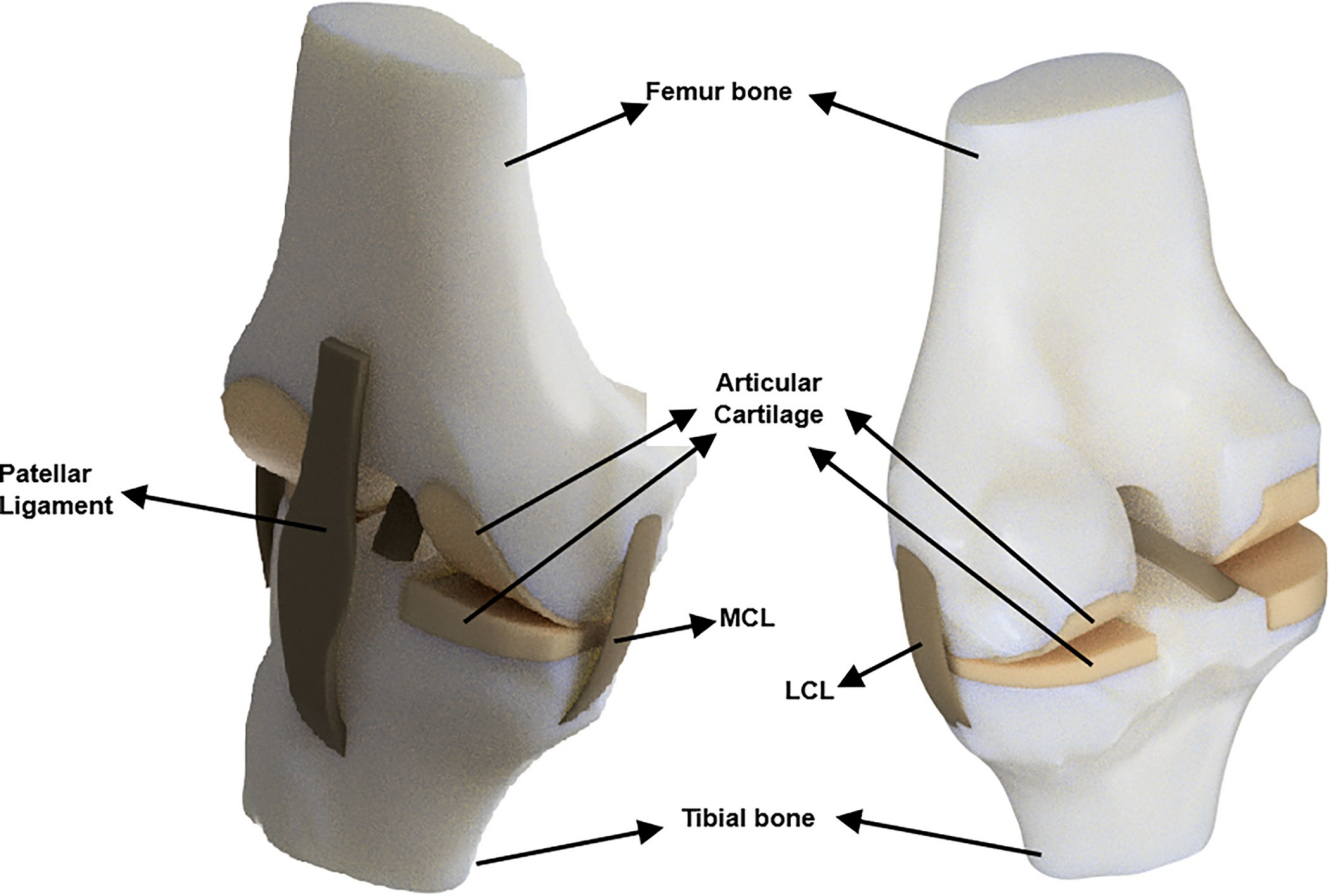
Final 3D solid knee model with nomenclature.

The model developed (in. STL) is not viable for nalysis and hence converted to a solid computer aided design (CAD) model. To achieve this, the model was transformed into the surface modes and stitched together. This design process was conducted using CATIA software. Subsequently, a solid CAD model was created using Autodesk Fusion 360, as illustrated in Fig 2. The 3D-rendered model of the knee joint, as depicted in Fig 2, served as the basis for developing the implants.

The model was rendered and color-coded to provide a realistic appearance and visual distinction of the anatomical components. Later, the solid model was further prepared for the dynamic analysis in ANSYS (2022 R1). The material properties of the knee joint bones and ligaments used for the analysis were chosen based on the literature [14, 31–35, 43, 44] as listed in Table 1.

**Table 1:** Material properties of the knee joint bones and ligaments [14, 31–35, 43, 44].

Parts	Material properties		
	Density (kg/m^3^)	Young’s modulus (MPa)	Poisson’s Ratio
Femoral part-bone	1900	18600	0.3
Articular Cartilage-femur	500	123	0.35
Articular Cartilage-tibia	500	123	0.35
Tibia-bone	1900	18600	0.3
Medial Collateral Ligament	1300	425	0.4
Titanium	4500	110000	0.35
Polyethylene spacer	950	20	0.4

### 2.3 Components of total knee joint implant

**Fig 3:**
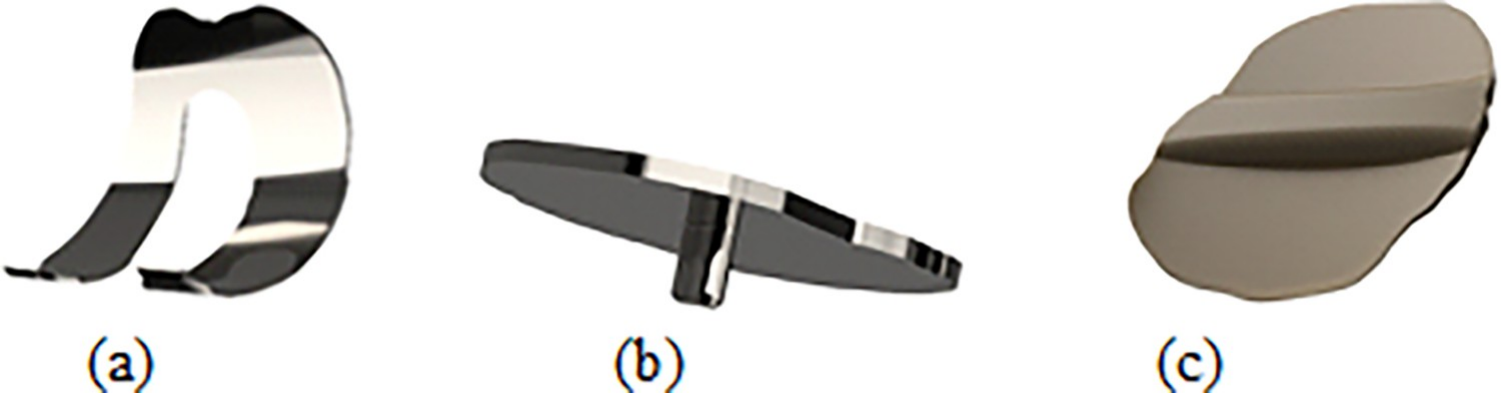
Components of knee joint (a) Femoral component; (b) Tibia component; (c) Polyethylene spacer.

Fig 3A illustrates the implant employed to replace the femur bone’s articular cartilage. Titanium (Ti) was chosen as the material for the articular cartilage replacement due to its favourable characteristics, including its relatively lightweight, elastic nature, and biocompatibility [35]. The strong mechanical properties of titanium provide an advantage in mitigating wear between the moving components during dynamic activities performed by the patient. The titanium replacement was mated with the polyethylene Spacer. Fig 3B shows the model of the tibia component. The tibia bone was cut to a recommended level and a titanium tibia component was attached to it. The surgeon generally cuts 5–10 mm and shapes the bone to fit the implant. In the present case, the bone mass was removed to accommodate the custom-designed implant. Fig 3C illustrates Polyethylene spacer which is one of the most prominent components of the knee joint is placed in between the tibia and femoral components. The polyethylene spacer component ensures the smooth sliding of the femoral component, preventing direct contact with the tibia component and thereby reducing the risk of abrasion. The polyethylene spacer material has been demonstrated to possess excellent longevity and enhanced wear resistance [36]. Based on our review, it is seen that the implants by manufacturers have their version of preserving a few ligaments and replacing a few, and no one implant can be said to be superior to another. So, in this implant design the articular cartilages, LCL, and MCL are preserved, and the simulation was done including this and excluding the other ligaments.

### 2.4 Model analysis

**Fig 4:**
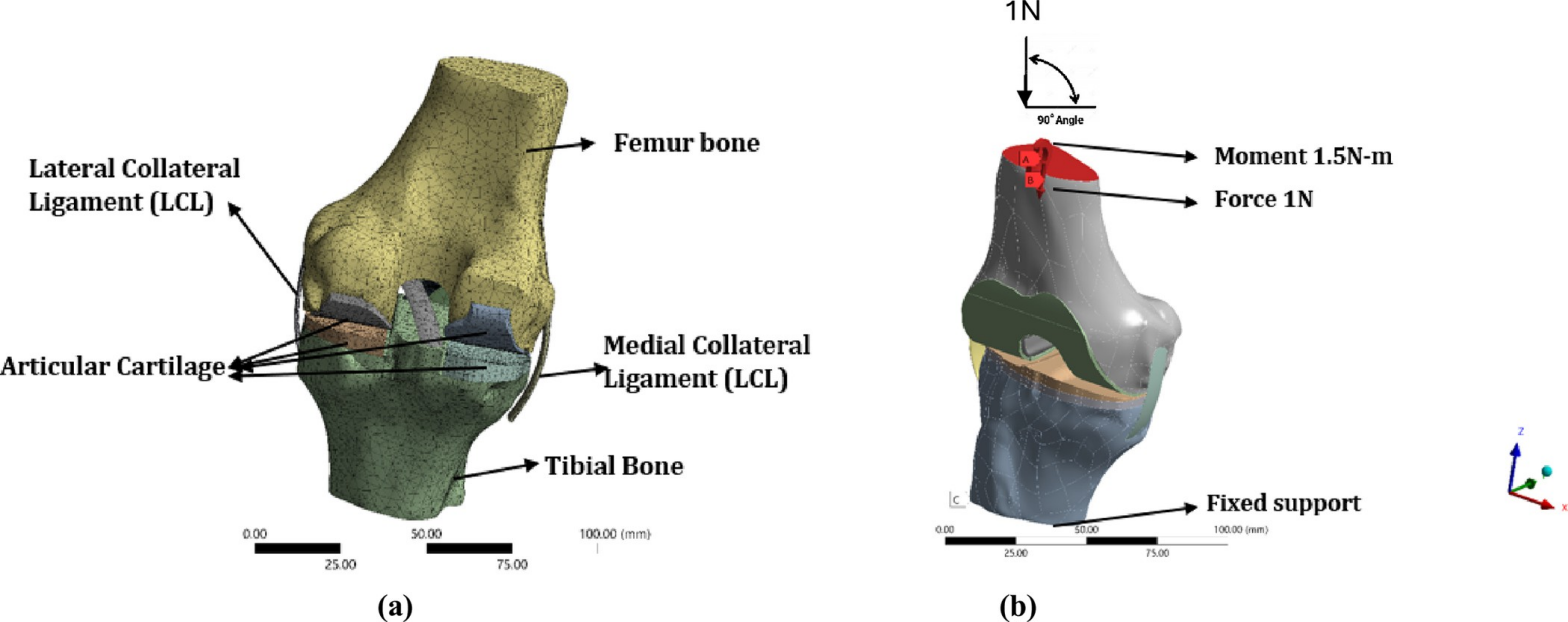
Solid Model (**a**) Tetrahedron mesh model; (**b**) Constraints incorporated mesh model.

A dynamic analysis of the developed model was performed to determine the deformation, stress, and strain under various loads and moments. The analysis was performed on he knee implants by considering the force of 750 N and 1000 N and moment of 0.8 N-m and 1.500 N-m, respectively. The various constraints and conditions are specified in the model and meshing was conducted by using tetrahedron elements. Before conducting the analysis, the constraints were specified and very fine meshes were utilized to ensure precise analysis results. The analysis was performed using ANSYS workbench version 2022R1 static structural platform where the deformation, strain, and stress were evaluated under forces of 750 N and 1000 N along with the moment of 0.8 N-m and 1.500 N-m, respectively. The tetrahedron mesh model is shown in Fig 4A while Fig 4B shows the meshed model with incorporated constraints. Tetrahedron meshes are chosen as they can very well adapt to complex surfaces. The current mesh count arrived after performing a mesh sensitivity analysis and the present count was adopted once the convergence was reached. Owing to space constraints we have not presented the results for mesh sensitivity. The final mesh count has been specified in Table 2.

**Table 2:** Comparison of results with and without implant.

Analysis type	Mesh Details	Parts	Results		
			Deformation* (mm)	Stress* (MPa)	Strain (10^−3^)
Without Implant Weight: 100–105 Kg. Force: 1000N. Moment: 1.5 N-m	Tetrahedron Elements (Nodes: 76197; Elements: 43009)	Femoral part-bone	5.62	24.53	1.35
		Articular Cartilage-femur	4.11	28.17	273.16
		Articular Cartilage-tibia	0.37	28.17	140.00
		Tibia-bone	0.54	18.87	135.00
		Medial Collateral Ligament	1.67	44.11	104.00
		Lateral Collateral Ligament	3.35	57.34	118.00
Without Implant Weight: 75–80 Kg. Force: 750 N. Moment: 0.8 N-m	Tetrahedron Elements (Nodes: 76197; Elements: 43009)	Femoral part-bone	4.37	18.99	1.04
		Articular Cartilage-femur	3.18	21.82	211.00
		Articular Cartilage-tibia	0.42	12.29	42.30
		Tibia-bone	0.02	30.28	6.12
		Medial Collateral Ligament	2.59	44.46	19.50
		Lateral Collateral Ligament	1.29	34.28	81.20
		Femoral part-bone	4.37	18.99	1.04
With Implant Weight: 100–105 Kg. Force: 1000N. Moment: 1.5 N-m	Tetrahedron Elements (Nodes: 83437; Elements: 47452)	Femoral part-bone	0.01	23.38	0.12
		Articular Cartilage-femur	0.01	25.97	0.15
		Articular Cartilage-tibia	0.002	29.86	0.16
		Tibia-bone	0.002	15.74	0.08
		Medial Collateral Ligament	0.002	14.80	0.07
		Lateral Collateral Ligament	0.005	64.74	0.34
With Implant Weight: 75Kg-80 Kg. Force: 750N. Moment: 0.8 N-m	Tetrahedron Elements (Nodes: 83437; Elements: 47452)	Femoral part-bone	0.008	17.98	0.09
		Articular Cartilage-femur	0.005	20.01	0.12
		Articular Cartilage-tibia	0.001	23.10	0.12
		Tibia-bone	0.001	12.17	0.06
		Medial Collateral Ligament	0.001	11.47	0.06
		Lateral Collateral Ligament	0.004	50.16	0.26

* All units are in SI.

## 3. Results

Analysis of customized knee implants based on patients’ anatomical images using FEA analysis in different loading conditions is presented in Table 2. Parts of the knee joint and mesh details used for analysis are also listed. This analysis was done on the knee of the specific set of patients with 100–105 kg: 75–80 kg of their body weight. Consequently, the force applied to the knee is determined by converting the body weight, enabling the analysis of its impact on the knee’s behavior. In addition to the applied force, a moment is also exerted on the patient’s knee. This moment is applied to simulate the actual motion of the knee, allowing for the analysis of deformation and strain rates on the surfaces of each component.

### 3.1 Comparative study

#### 3.1.1 Comparison of results among the patient’s weight (100–105 kg vs 75–80 kg)

Table 2 indicates that patients with higher weight exhibit greater values of deformation, stress, and strain. As individuals age, factors such as decreased bone density, reduced toughness, and various health-related issues can contribute to these changes. Consequently, patients with higher body weight are more likely to experience increased wear on their cartilage, which can impact the positioning of the knee and lead to ligament tears. Even in healthy individuals, greater weight can exert an influence on cartilage wear. In the analyzed patient, a twist in the tibia bone was observed, resulting in an effect on the corner portion of the tibia, including the cartilage, due to the applied force. Therefore, maintaining a healthy weight is crucial.

The alignment and wear of the knee joint are influenced by numerous factors. This study specifically focuses on the influence of body weight on the knee. The results from the patients with implants further indicate that patients with higher weights experience significantly higher levels of deformation, stress, and strain compared to those with lower weights. Consequently, the strain value serves as a warning sign of substantial component deterioration.

#### 3.1.2 Comparisons of the results between the patient with and without implant

Table 2 demonstrates the significance of the patient’s implanted knee joint, as it exhibits lower levels of stress, deformation, and strain compared to the un-implanted knee joint. The utilization of titanium implants offers protection to the femur and tibia bones by limiting excessive deformation and mitigating elevated stress levels. Furthermore, the femoral component, which moves smoothly over the polyethylene spacer, experiences lower strain rates compared to other regions. Consequently, the proposed implants play a crucial role in safeguarding the knee joints from deterioration, ensuring their overall success. Furthermore, due to reduced deformation, stress, and strain in the knee joint with implants, the impact on the MCL and LCL is significantly minimized. The findings depicted in Table 2, which showcase diminished stress, deformation, and strain in the implanted knee joint when compared to the unplanted knee joint, provide validation for the anticipated advantages of the proposed implants. By utilizing titanium implants, the femur and tibia bones are shielded against excessive deformation and stress. Furthermore, the observation of lower strain rates in the femoral component gliding over the polyethylene spacer signifies the successful performance of the implants. Additionally, the diminished effects on the MCL and LCL suggest that the implants effectively reduce stress levels and enhance knee movement.

The study serves as a general analysis for a specific patient population. However, it should be noted that these analyses can be personalized to individual patients since each person has a distinct body shape, gender, ethnicity, and the potential for hereditary traits. Consequently, a comprehensive effort and extensive data are necessary to thoroughly investigate and draw general conclusions. As a result, a vast collection of data sets must be analyzed to identify and generalize suitable implants for different individuals.

### 3.2. Structural analysis of the knee joint

**Fig 5:**
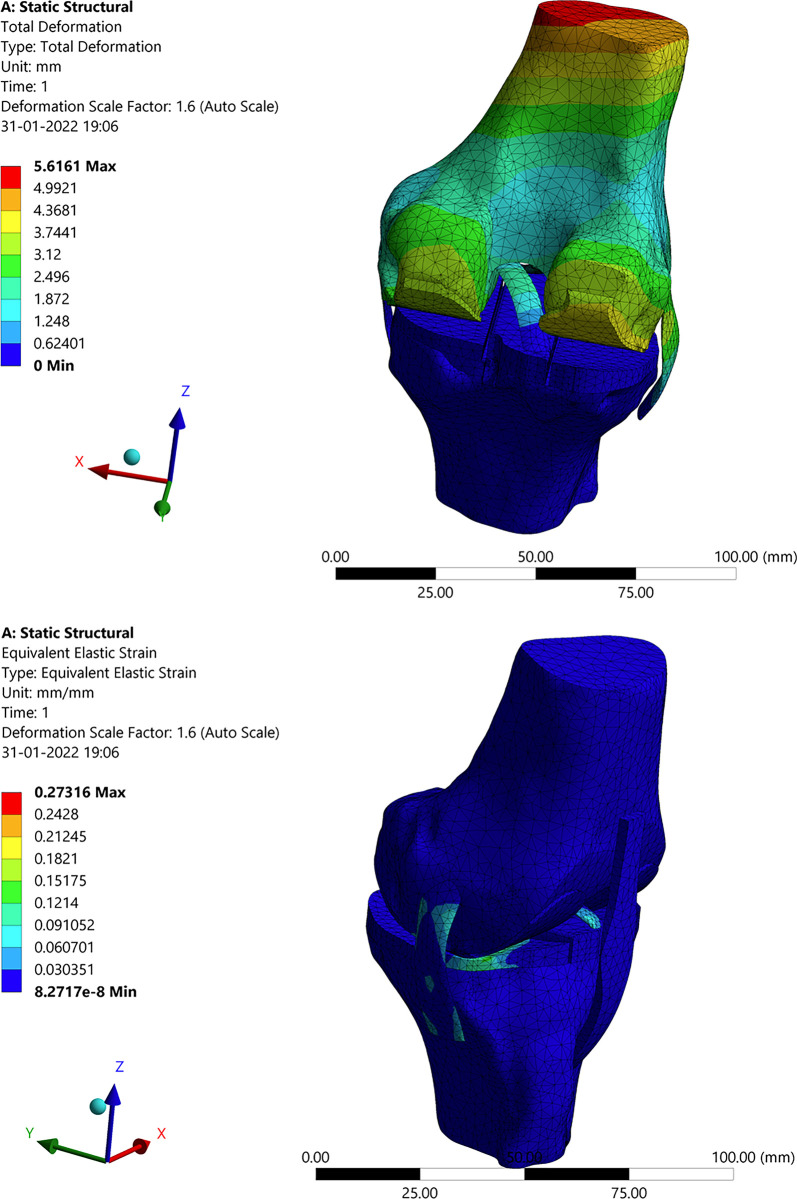
Results of the structural analysis: (**a**) Total deformation of knee joint; (**b**) Total stress of knee joint.

The model is analyzed to examine the effects of the applied load on deformation, stress, and strain. Fig 5 depicts the knee joint’s overall deforestation, stress, and strain. Moreover, it presents the maximum and minimum values of deformation, stress, and strain. A specific set of patient weights, such as 100–105 kg and then 75–80 kg, were used for the analysis. Accordingly, a patient weighing 100–105 kg is subjected to a force of 750 N and a moment of 0.8 N–m, and a patient weighing 100–105 kg is subjected to a force and moment of 1.0 N–m and 1.500 N–m, respectively. The data for each articular cartilage, ligament, femur bone, and tibia bone are presented in Table 2 and subsequently discussed. These findings are then compared and analyzed in relation to the results obtained from knee joints with implants.

#### 3.2.1. Deformation, stress and strain in femur bone

**Fig 6:**
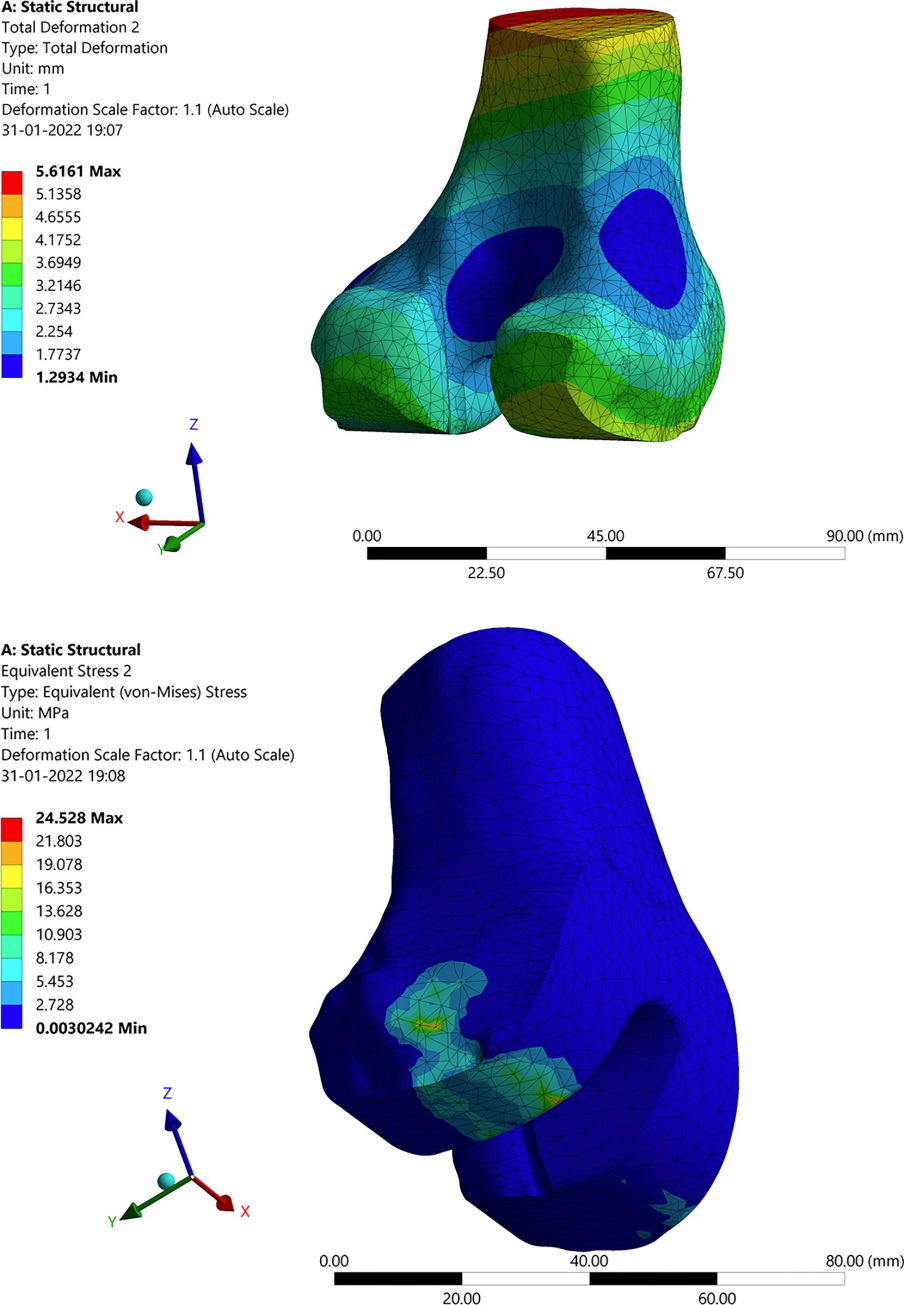
Structural analysis: (**a**) Deformation in femur bone; (**b**) Stress distribution in femur bone.

In Fig 6, a structural analysis of the femur bone, connecting the knee joint, is presented. The analysis investigates the response of the individual components of the femur bone to an applied load of 100–105 kg, simulating body weight. This analysis aims to understand the behavior of the femur bone under specific force and moment conditions. The maximum and minimum values for deformation, stress, and strain are shown in Fig 6A and 6B. In contrast to the overall deformation of a specific femur bone, the highest stress and strain levels have been observed to occur in the medial positions. This is attributed to the stabilizing effect of the medial collateral ligament on the knee, which helps offset the deviation caused by the applied force and moment.

#### 3.2.2. Deformation, stress, and strain in articular cartilage attached to the femur

**Fig 7:**
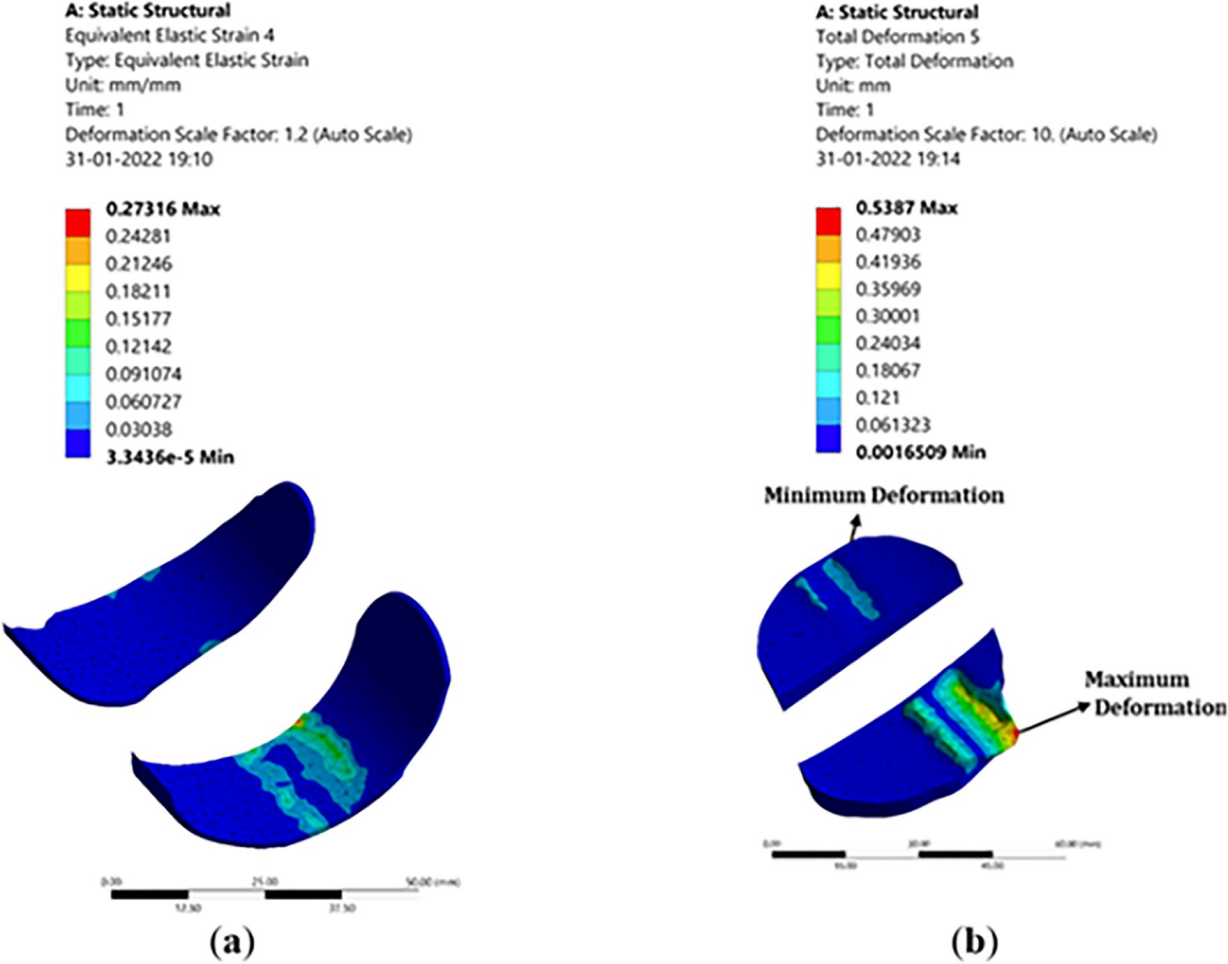
Structural analysis: (**a**) Stress in articular cartilage attached to the femur; (**b**) Deformation in articular cartilage attached to Tibia.

Similar to the femur bone, the deformation, stress, and strain are transmitted to the articular cartilage. In this process, the cartilage attached to the femur slides over the cartilage attached to the tibia, mitigating the impact. However, due to this sliding action, the cartilage experiences wear, as illustrated in Fig 7A. Ligaments serve as stabilizers to facilitate controlled movement. In Fig 7B, the highest levels of strain, deformation, and stress are observed at the corners of the cartilage. This is primarily attributed to the disorientation and increased impact from the femur, resulting in greater wear and stress concentration at the corners of the cartilage.

#### 3.2.3 Deformation, stress, and strain in tibia

**Fig 8:**
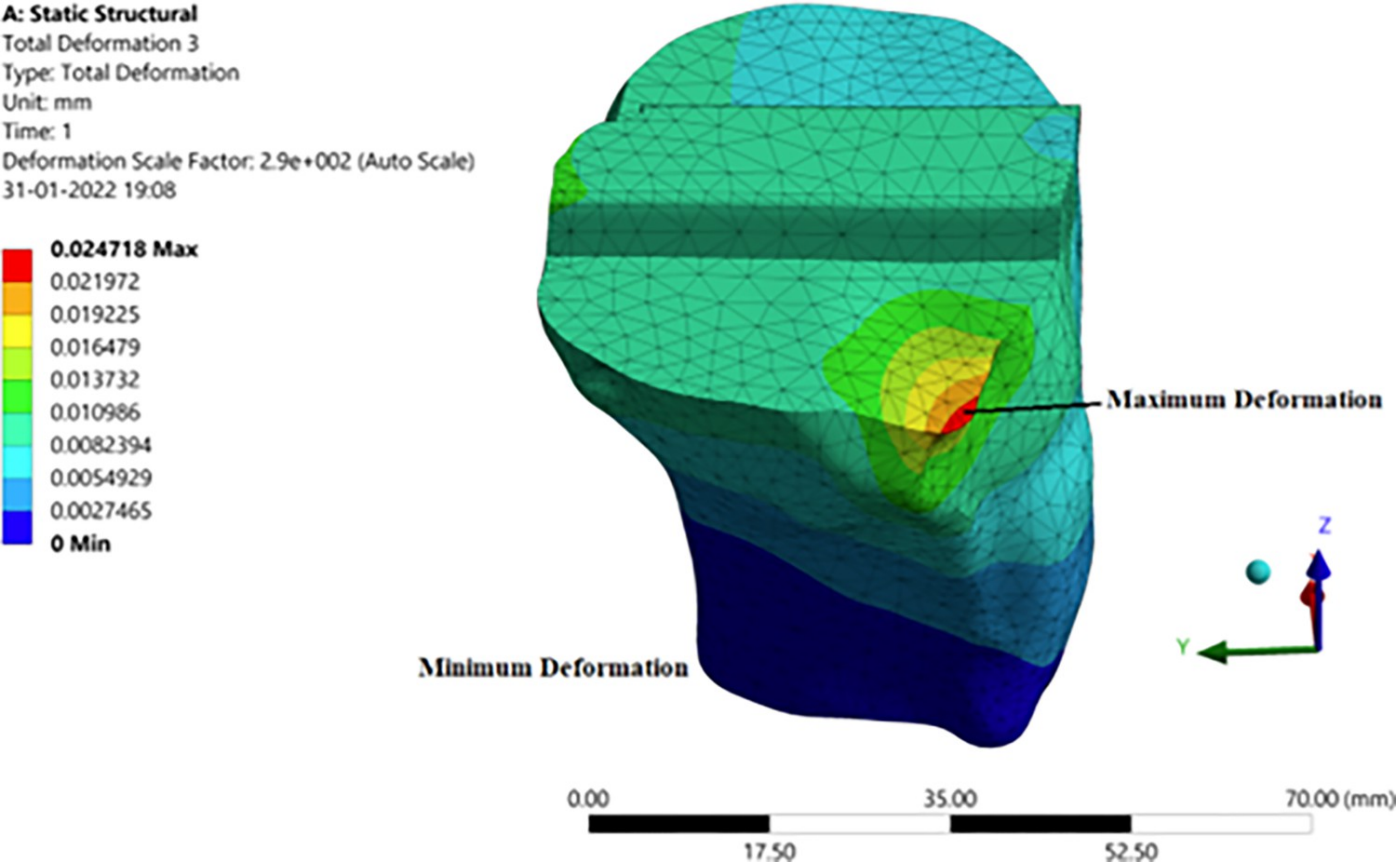
Structural analysis: Deformation in Tibia.

Unlike the tibia cartilage, the femur has more impact on the tibia corner also and can be seen in Fig 8. There is a maximum intensity of stress developing on the corner of the tibia due to the load acting on the femur.

#### 3.2.4 Deformation, stress, and strain in medial collateral ligament (MCL) and lateral collateral ligament (LCL)

**Fig 9:**
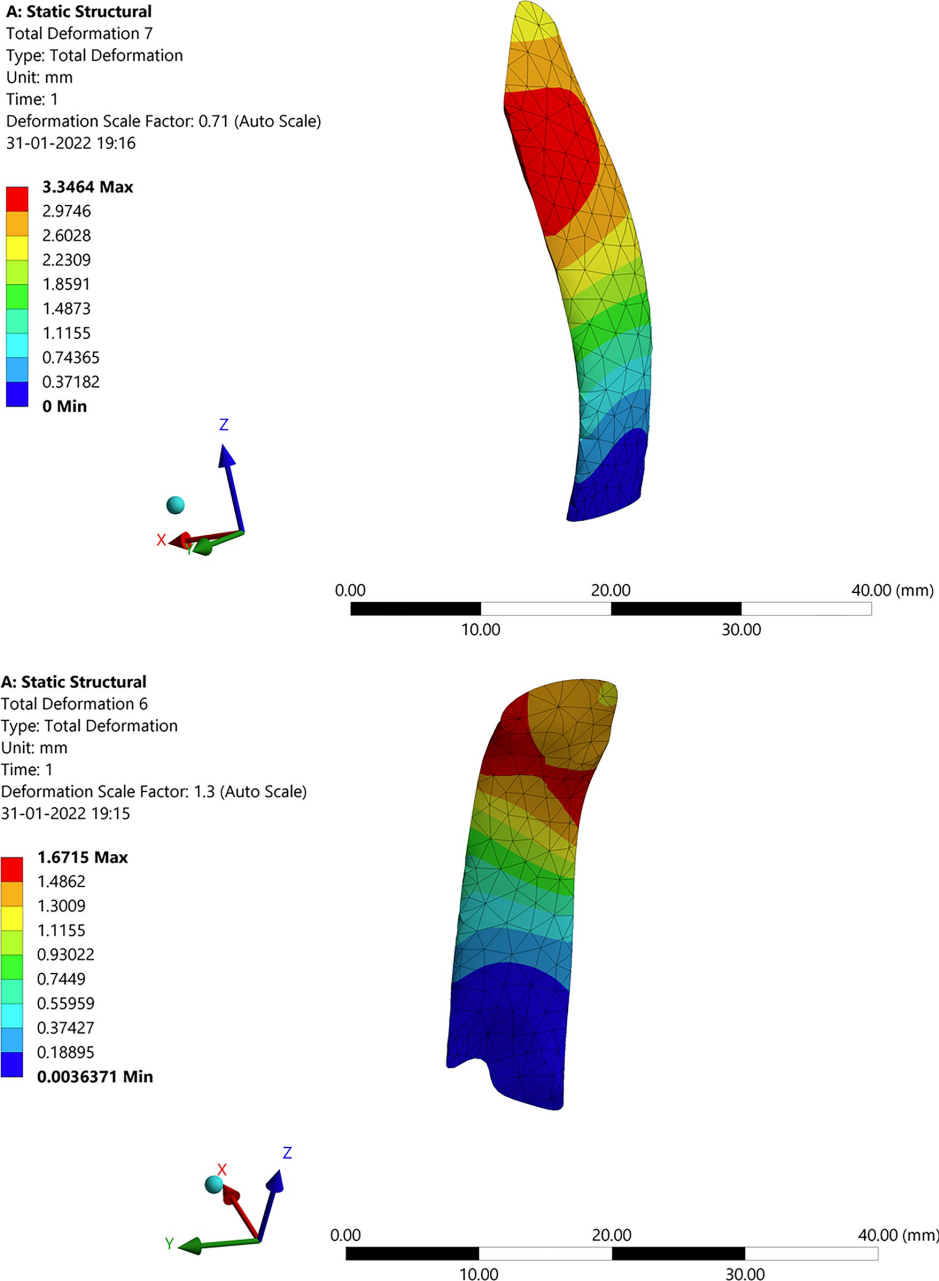
Structural analysis of the ligaments: (**a**) Deformation in MCL; (**b**) Deformation in LCL.

As the other components, the ligaments play an important role in stabilizing the knee joint by bringing them or keeping them intact during hyper actions or the stress developed on it. MCL and LCL are the main types that keep the joints in proper position. MCL is at the left of the knee and attached to the tibia, LCL is at the right and attached to the fibula. The analysis results are shown in Fig 9A. Here it can be seen from Fig 9B that both MCL and LCL have high intensity of deformation, stress, and strain at the point of contact itself. That is at the point where MCL and LCL get connected to the femur bone. This is due to both force and the moment that is acted upon the femur which as a result affects every other component.

### 3.3 Structural analysis of the knee joint with an implant

**Fig 10:**
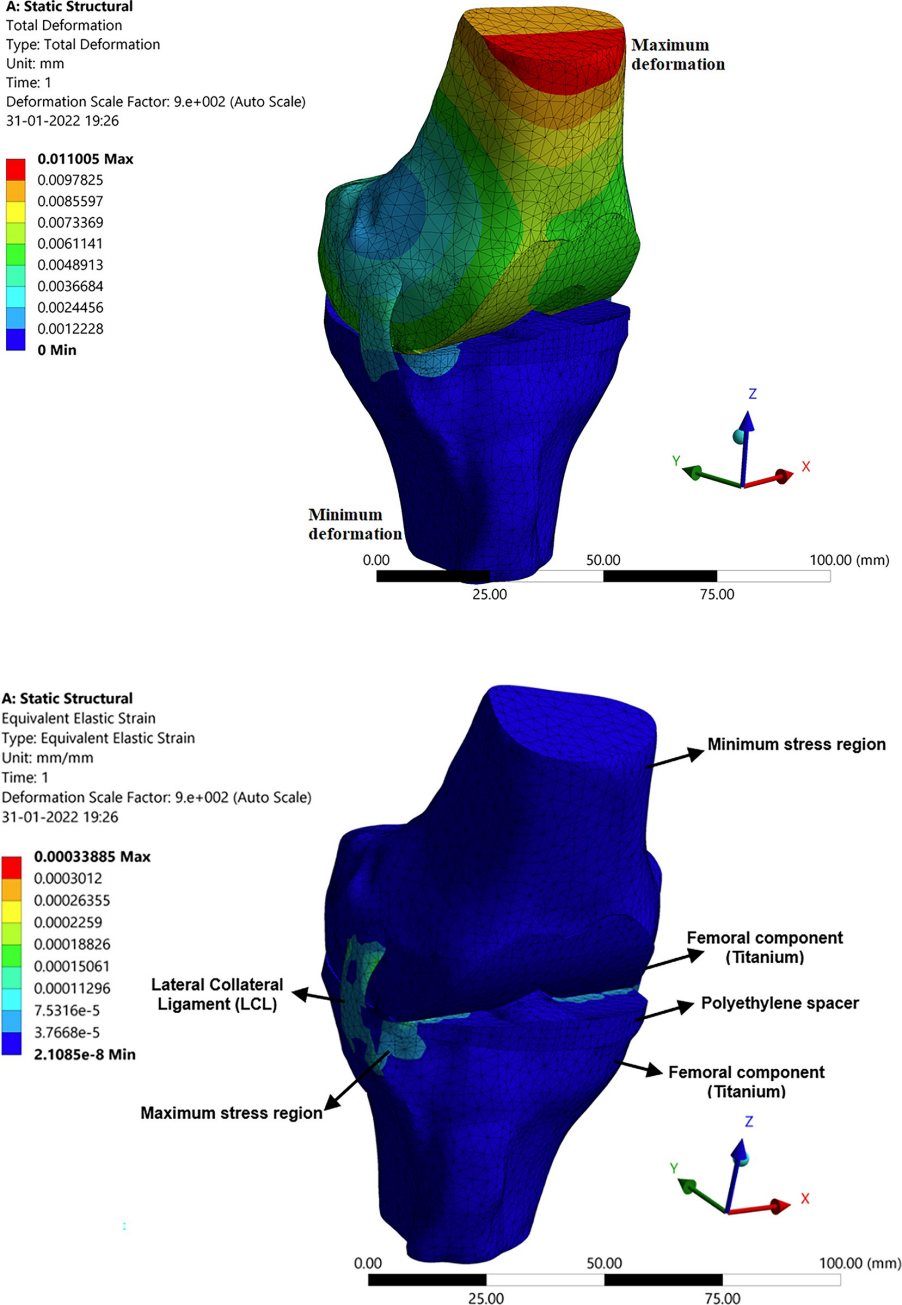
Structural analysis with implant: (**a**) Total deformation of the knee joint; (**b**) Stress in the knee joint.

The total deformation, stress, and strain for the knee joint with implants were analysed in the developed model with the above-mentioned constraints, and the results are presented here. Fig 10A shows the total deformation obtained because of the applied force of 1000 N and moment of 1.500 N-m. In this figure, the colour pattern represents the intensity of the values obtained. The red colour pattern shows the high-intensity value, which is maximum deformation, and the blue colour pattern yields mini-mum deformation. As a result of the deformation, the stresses, and strains are developed between the mating surfaces between the knee joint implants. Since the femoral component which is a titanium implant for the femur slides over the polyethylene spacer. This polyethylene spacer is mounted upon the tibia component. Due to the compressive force and the acting moment on the femur, these stresses and strains are developed. Fig 10B shows the stress and strain distribution in the implants of knee joints. This is due to the result of the effective moment and compressive force on the femur bone. Since the tibia bone is fixed the load (weight of the human body) falls upon the tibia portion wherein the polyethylene spacer absorbs, and stresses are developed in this region as a result strains are developed. The figure mentions the maximum and minimum intensity values and the colour pattern legend defines every corresponding value.

#### 3.3.1 Deformation, stress, and strain in the femur bone

**Fig 11:**
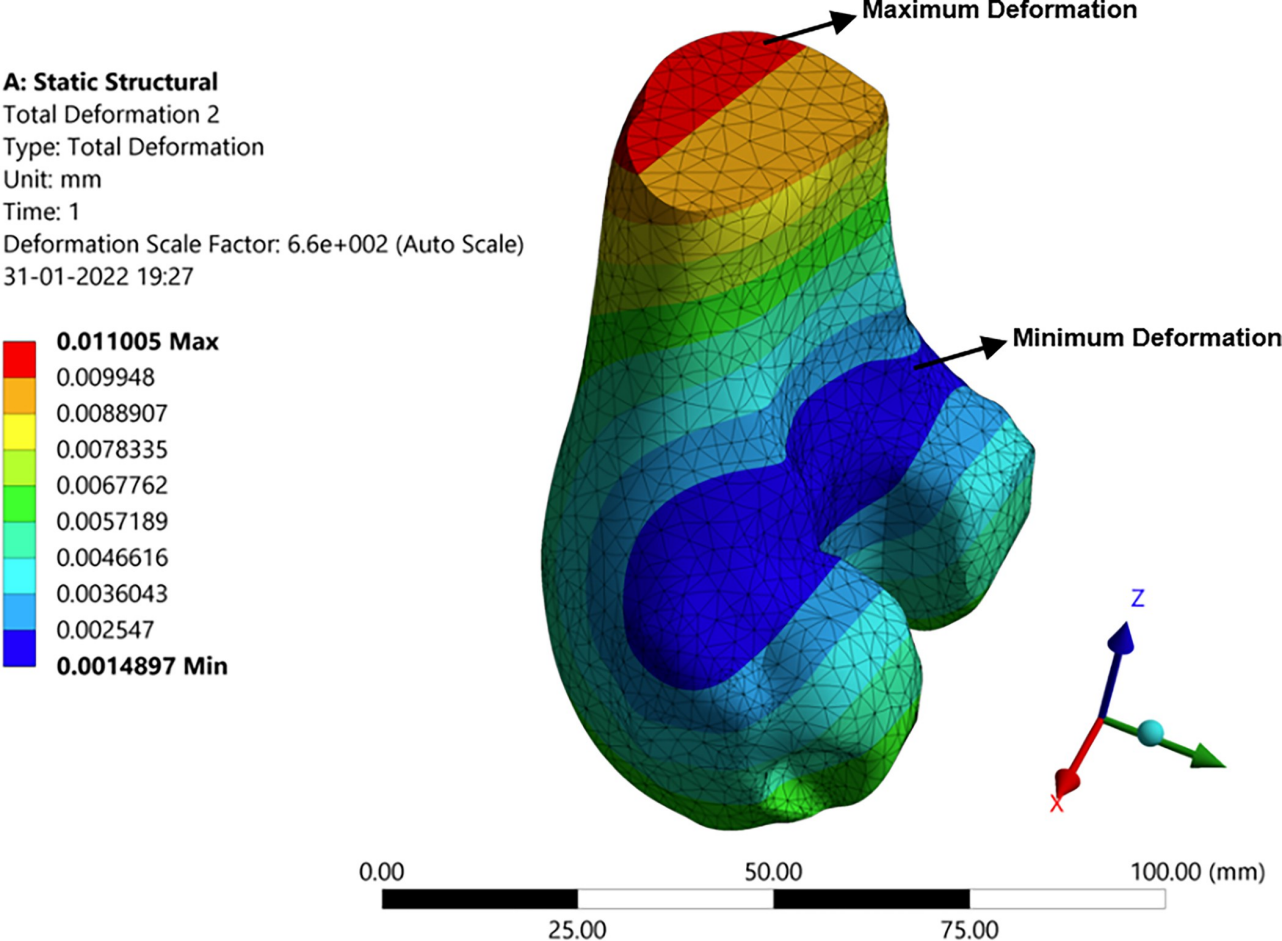
Structural analysis with the implant in femur bone.

This section shows the structural analysis results of the individual component to know how each component behaves in contact with others when the constraints are applied to it in totality. Fig 11 shows the deformation, stress, and strain distribution on the femur bone because of the compressive force and the moment of 1000 N and 1.5 N-m respectively. The maximum and minimum values are mentioned in the legend via colour patterns. The colour patterns on the femur bone also signify how the deformation, stress, and strain at each point are in effect and varied accordingly. The material properties are tabulated in the tabular column in the previous section. The stress and strains are developed between the femoral component which is made up of titanium which the femur bone is attached to.

#### 3.3.2 Deformation, stress, and strain in articular cartilage attached to the femur

**Fig 12:**
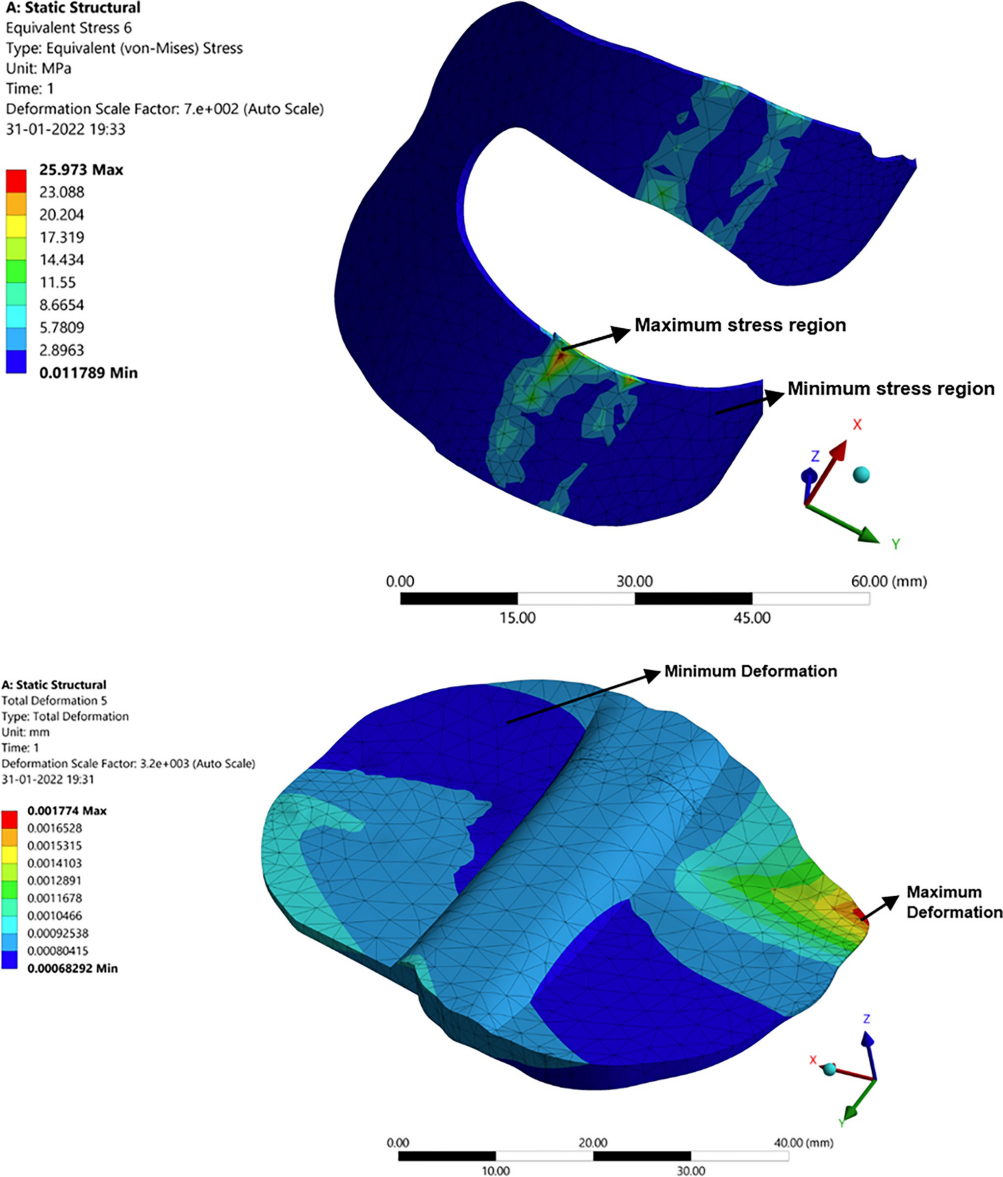
Structural analysis with implant: (**a**) Stress in femoral component; (**b**) Deformation in polyethylene spacer.

The femoral component made up of titanium is attached to the femur bone in the place of articular cartilage. It is generally used to protect the femur bone from wearing further and is designed as per the femur bone profile using the Fusion 360 software. This implant acts as articular cartilage by sliding on the polyethylene spacer with improved wear resistance. Fig 12A show the representation of deformation, stress, and strain signifying the effect of forces on the femur which further adds up to the component. As we can see the deformation is high at the tip of the femoral component as the couple acts on it. The intensity of the stress and strain are more at the edges and are nominal since there is no sign of significant wear of the component. This is because the femoral component is in contact with the femur bone and the polyethylene spacer, hence the stress will be more at this junction which results in larger strains.

Polyethylene material is widely used for implants due to its biologically inert property, also non-biodegradable in the body. The porosity of high-density polyethylene allows for soft tissue and vascular growth, which helps in keeping the implant in place. These materials have improved resistance to wear and are indestructible. Due to the indestructible property polyethylene materials are widely recommended for implants. Fig 12B shows the deformation, strain, and stress distribution observed in polyethylene spacer. The compressive force on the femur acts on the polyethylene spacer enduring deformation in the polyethylene spacer. The femoral component slides over the polyethylene spacer at the corner due to the twist in the femur bone or the tibia bone. Hence the stresses are more at the corner of the spacer about a large strain value. Hence, it’s recommended to re-orient the bone forms to their regular orientations. Also, it may be noted that the properties of bone, orientation, and size will matter for different age groups, different genders, and different ethnicities. Hence all the analysis will be patient specific.

#### 3.3.3 Deformation, stress, and strain in the tibia component

**Fig 13:**
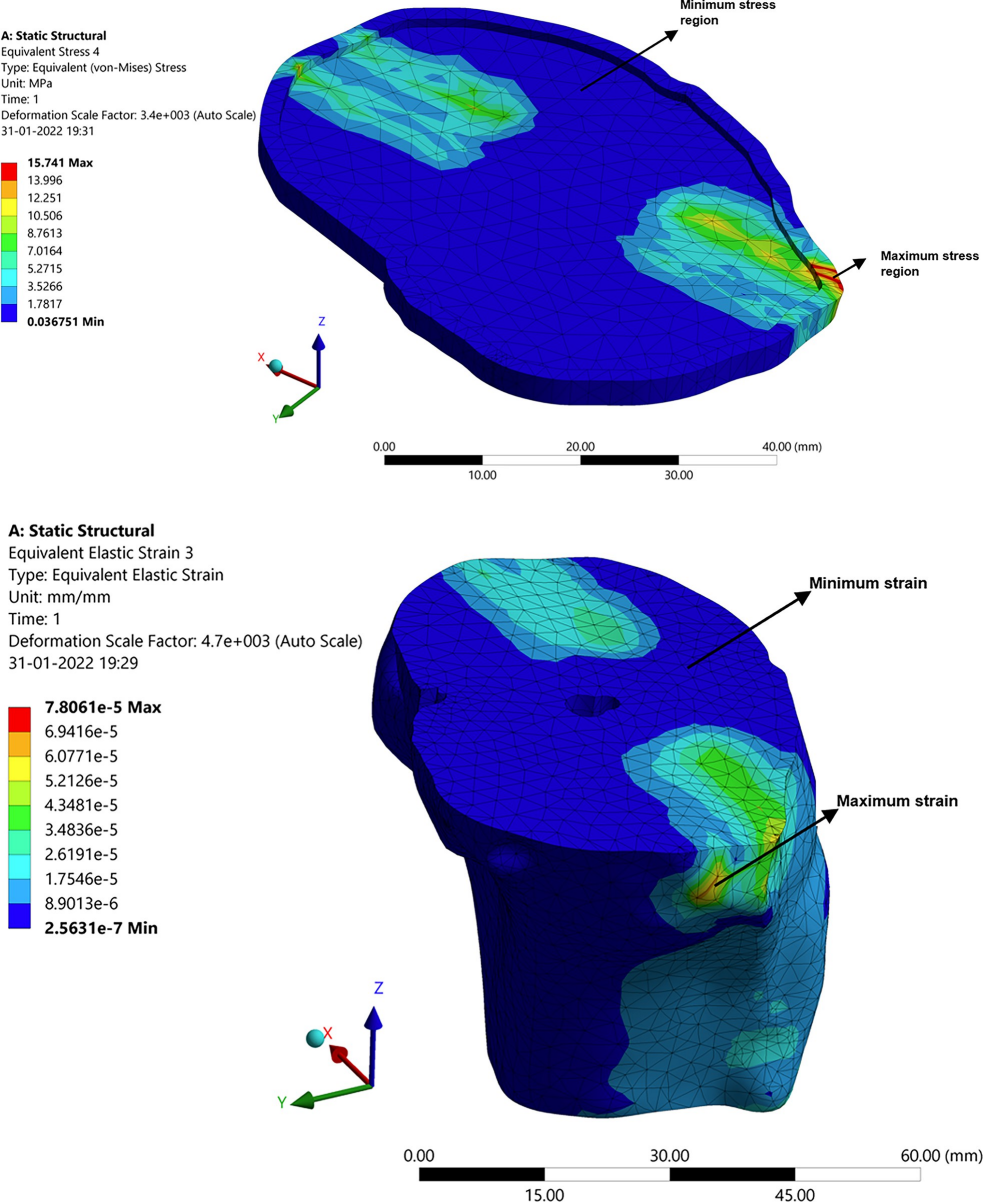
Structural analysis with implant: (**a**) Stress in tibia component; (**b**) Deformation in tibia.

The tibia component is a titanium component which is supporting the polyethylene spacer to have a proper orientation. The tibia bone is cut to a certain level by the surgeon who drilled a hole on it to support the tibia component. The results of the analysis are obtained which are discussed below. It can be seen from Fig 13A that larger deformation and stress in the corner and at the edge. This is because of the disoriented bone. There is more pressure at the corner of the tibia component since the femur slides upon it in the corner. It is known that the tibia portion is the one that supports the body and enhances the leg momentum. It’s the same material as that of the femur with similar properties. Due to the locomotion, the polyethylene spacer and femoral component will be in frequent abrasion. The various stress and strain on the tibia can be observed in Fig 13B. The stress runs through all over the tibia pertaining same as that of deformation. The deformation on the tibia will be less when compared to the femur since the forces on the femur will be absorbed or cancel out at the femoral component and polyethylene junction through wear or sliding. Since the lateral deformation will be less when compared to that of the longitudinal deformation, the strain levels are nominal. Hence the tibia can successfully hold the weight of 1000N on it.

#### 3.3.4 Deformation, stress, and strain in medial collateral ligament (MCL) and lateral collateral ligament (LCL)

**Fig 14:**
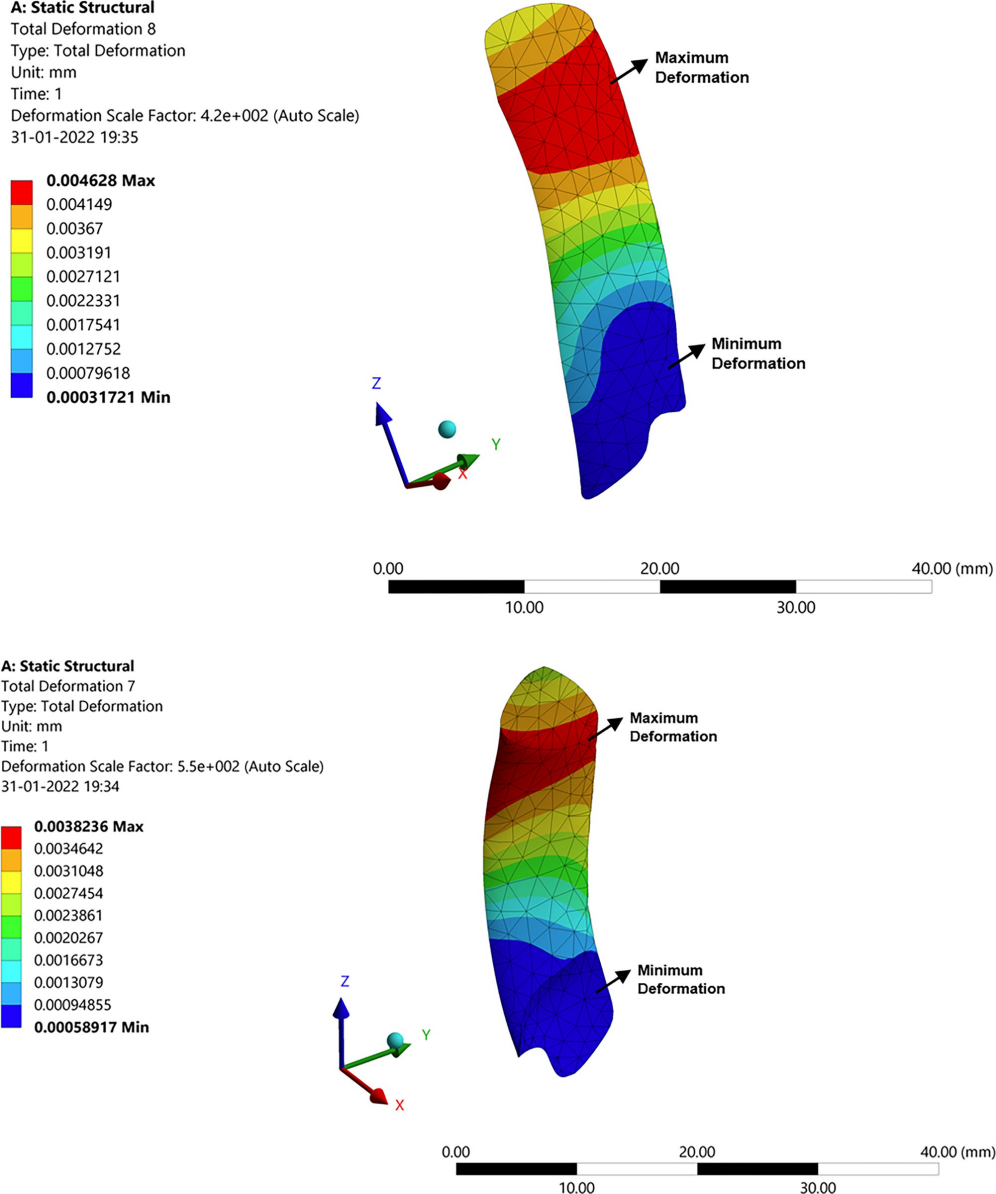
Structural analysis with implant: (**a)** Deformation in MCL; (**b**) Deformation in LCL.

The main function of MCL is to prevent the leg from stretching/extending too far inward. It also helps the knee to be at a stable orientation and allows it to rotate. MCL has the greatest healing capacity since it is also injured so easily. The MCL is on the left side of the knee, and it connects the femur to the tibia. It has a greater significance in the analysis which is why it must be considered for the analysis. The pictorial representation for the analysis is shown in Fig 14.

The MCL and LCL are included in the study primarily because they join the femur to the tibia. As a result, the tibia or femoral motion has a relative impact on the activity. This part regulates the motion and prevents the knee from moving outward. The colour patterns for the various deformation values may be seen in Fig 14. The femoral location exhibits the highest levels of deformation, while the margins of the MCL exhibit the highest levels of stress and strain. This is because of the tensile tension that the MCL experienced because of the femur bone moment. The LCL performs the same job as the MCL in regulating knee motion, assisting in maintaining knee stability and enabling rotation of the knee. It is situated on the right side of the knee and joins the femur to the fibula. LCL is just as significant in the study and has comparable importance as MCL. At the borders of the LCL, where deformation and stress are most pronounced, stresses arise as a result. Therefore, the LCL and MCL are not now experiencing a harsher impact. We may deduce from the study that the MCL and LCL function as stabilizers of the knee, providing both medial and lateral stability as well as some degree of rotational stability. These elements help to maintain the knee stable and in place.

## 4. Discussion

The key differentiating factor of our customized total knee implant lies in its ability to replicate realistic loading conditions using FEA. By accurately simulating the forces and stresses experienced by the knee joint during various activities, we can optimize the implant’s design and ensure optimal fit, stability, and longevity. This approach allows us to consider the unique characteristics of each patient, considering factors such as bone density, alignment, and soft tissue interactions. The entire process is the load absorption and distribution. For example, the dense bone would carry the entire load, and the soft tissues would compress and absorb the load. The implant customization by shape will help in proper load distribution, thereby providing better comfort to the patient. Similarly, choice of material will help in absorbing part of the load by compression and reduction of friction during mobility. The analysis performed in this work substantially supports patient-specific customized knee implants for the Saudi Arabian population than conventional on-the-shelves (OTS) implants. For various body weights, the deformation, stress, and strain values of the knee joint with and without implants were calculated and compared.

In this work, boundary conditions are applied to the model as the Tibia bone has been constrained in all 3 axes and the load is applied in Z- the axis on the femur by keeping the other two axes constrained. This gives a more realistic approach to understanding the wear and tear of the cartilage for the model without implants and polyethylene spacer with implants. This helps to analyze the impact of load distribution, stress concentration, soft tissue integrity, and joint stability of the model with and without implants.

Unlike the total deformation for a particular femur bone, the maximum stress and strain level have been shifted to the medial positions as the analysis carried out earlier [34, 35]. This is because the medial collateral ligament stabilizes the position of the knee where the deviation is incurred due to the force and the moment applied to it. The maximum strain, deformation, and stress can be seen at the corner of the cartilage and is because the femur has been disoriented and has more impact and wear on the corner of the cartilage. This analysis was not found in earlier researchers [36, 37]. Our finding also supports the analysis carried out by Dupraz et al. [2022] in comparing the impact of femoral-tibia TKR design on kinematics [38].

In this work, the 3D modeling approach to realize the customized knee implant for the Saudi Arabian population has been analyzed by considering its kinematic parameters, and its performance has been improved and optimized in post-implant conditions compared to earlier studies [39–44]. The study aims to deliver a customized total knee implant that closely mimics the natural knee joint mechanics for the Saudi Arabian population, promoting better load distribution, reduced wear and tear, and improved patient satisfaction. By incorporating articular cartilages into the implant design, we strive to restore the natural functionality of the knee, minimizing pain, and enabling patients to regain their mobility and quality of life. Through our findings, it can be ascertained that knee dynamics can be improved in post-customized implant conditions with some practical limitations and challenges. In this study, only validation studies have been done to prove the robustness of the FEA in customized knee modelling and no clinical trials have been performed. Obtaining big data sets of the patient’s CT scans with several slices will enable further model optimization. This would be incredibly beneficial in creating simulations and models that are more accurate. Since ligament tears and cartilage deterioration in the knee occur more often, precise measures have to be taken care of while manufacturing knee implants. Although the simulation results are presented for one case, what has been aimed to present in this work is the culmination of CT, reverse engineering of the implant by CAD, analysis by FEA, and arriving at the optimum design. The implant could well address the lifestyle of the individual in terms of how the knee is used. In this case, we have assumed the Arab population and they have used kneeling/squatting postures during their regular prayers that could be well addressed before the model is printed for implant. As FEA tools are powerful, this could be easily incorporated during optimization of the design and has not been attempted too here and can be addressed in future.

The stress that is seen during the simulations can be limited or controlled by altering the design of the implant before printing the same. In case there is a limit to what can be achieved and controlled, no surgical approaches are recommended by us. However, as a part of post-operative rehabilitation the same needs to be communicated to the patient on limiting the posture or loading. This way there will be minimal surgical and physiotherapy intervention that would well offset the cost of initial modeling & analysis with customized 3D printing. Also, the customized implant would require less traumatic sizing of the bones making it convenient during and after surgery. The most effective rehabilitation and recovery protocols that should be followed by the implantation of this knee joint to guarantee the best possible outcomes for patients should also be considered in future studies. We have not made a dollar-to-dollar comparison. But personalization of the implants requires additional cost in terms of development to of three-dimensional code for printing, the cost would offset in terms of the intervention required in surgical procedure for fixing a customized implant than a readily available one. This reduction in surgical process as well as quicker recovery and lesser hospitalization would more offset the cost. The quicker recovery and improved quality of life cannot be quantified in dollar terms. Right now, cranial surgery uses customized implant for accident victims. Low costs options have been discussed in many literatures but not limited to these two papers [45, 46].

As the implants are custom made, we believe that this will also help in the outcome of patients having co-morbid illness as the customization reduces the surgical risks associated. However, we do acknowledge that we have not studied this aspect in the current work. The study’s future focus will extend to the creation of patient-specific knee joint implants for a wide range of patients. The choice between patient-specific and OTS or universal implants depends on factors like patient anatomy, clinical needs, and surgeon expertise. Patient-specific implants offer a tailored fit, potentially leading to better outcomes and reduced complications in complex cases. However, they are costlier and require additional processes. Universal implants are readily available, cost-effective, and familiar to surgeons. While they may lack the precise customization of patient-specific implants, they can still provide satisfactory outcomes in many cases. Ultimately, the decision should be based on individual patient considerations, surgeon preference, and available resources. Close collaboration between the patient and the surgical team is crucial in selecting the most suitable implant option. This we believe will enhance the life of the implant as well as the quality of life of the patient, as we do take care of lifestyle issues while designing the implant (as explained the Arabs have the habit of kneeling more often causing a different set of stresses than other population). This work can be further improvised by incorporating the post-clinical data to modify the boundary condition and reanalyze the fits and the performance of the implants on the patients. The average weight of the patient can be considered for future studies with larger data sets for normal distribution to perform better FEM studies. The use of modern numerical modeling techniques to enhance the quality of life of the patient in customizing the implant with presently used material is what is studied and presented here. Thus, this work is more on the improved design of existing materials rather than focusing on newer modern materials. This work could however be extended for newer modern material as suggested by the reviewer. An analysis of different implant materials that are biocompatible, flexible to manufacture, and low-cost can also be considered. However, the current work has not been studied over a period and the same is left for future scope. In the future, the entire process of feeding the CT scan to obtain the digital twin and further analysis for various loading can be fully automated. The output can then be fed to the 3D printer for getting the customized implants. From the data set so generated, artificial intelligence can then be built in to speed up the entire process for scalable and affordable implants with faster turnaround.

The difference in posterior tibia slope between Caucasian knees and those of Saudi and Asian populations is significant, primarily due to variations in habitual activities and physical postures such as squatting, kneeling, and deep flexion. Caucasian knees typically exhibit a lower posterior tibia slope, averaging around 7° to 9°. This slope is generally sufficient for activities that involve less extreme knee flexion. In contrast, Saudi and Asian populations often show a higher posterior tibia slope, which can range from 10° to 15° or even higher. This increased slope is an anatomical adaptation to the frequent performance of activities that require deep knee flexion, such as squatting and kneeling. These activities exert greater demands on the knee joint, necessitating a steeper posterior tibia plateau to accommodate the increased flexion and provide stability.

The higher posterior slope in Saudi and Asian knees plays a critical role in enhancing the range of motion and reducing the risk of anterior cruciate ligament (ACL) injuries during activities that involve deep flexion. However, this anatomical difference also means that standard knee implants designed for populations with lower tibia slopes may not be as effective for these groups. This underlines the importance of designing patient-specific knee implants that take into account the unique anatomical features and functional demands of different populations, ensuring better post-surgical outcomes and reduced discomfort. As elucidated in the paper on the effect of bush material property on the metal-polymer contact stress in knee megaprosthesis, an in silico study, FEA helps in clearly identifying regions of interest where the stress concentration is more and how interfaces region between different material properties (polymer & metal) affect the wear of the softer material, thus helping in the right choice of materials to minimise wear and tear [47]. So, customization in terms of anatomy and material properties are superior to existing methods.

## 5. Conclusions

The objective of this research was to validate the hypothesis that patient-specific customized knee implants enhance the post-surgical quality of life for patients within the Saudi Arabian population. The findings indicate that meticulously designed customized implants achieve a significant 5% reduction in stress levels. Central challenges and discussions in this field revolve around optimizing the treatment of knee osteoarthritis, improving implant performance, and minimizing post-surgical complications. By integrating finite element analysis (FEA) with patient-specific data, the proposed methodology introduces an innovative approach to comprehending knee joint mechanics and optimizing implant design. The deformation, stress, and strain values of the knee joint, both with and without implants, were calculated and compared across different body weights. These metrics contribute to a deeper understanding of joint strength and stability, underscoring the necessity of implants in compromised knee joints. The analysis incorporated ligaments and cartilage, crucial components of the study, as they play a significant role in joint mechanics. The findings suggest that wear is more pronounced in obese patients than in leaner individuals, highlighting the impact of body weight on implant performance.

Furthermore, the study reveals that knee-joint implants not only hold promise but also exert a positive influence on joint function. However, to thoroughly establish their efficacy and accurately gauge their benefits, further research and validation studies are warranted. The consideration of ligaments and cartilage, particularly the patella, was meticulously included in the analysis, with these components specifically modelled for the study. Additionally, the tibial component of the implant was precisely engineered to match the exact cross-sectional geometry of the tibia. We believe that our novel approach to customized total knee implants, incorporating articular cartilages under loading conditions via FEA, represents a significant advancement in orthopaedic technology for the Saudi Arabian population. The potential benefits of this innovation extend beyond improved patient outcomes, promising shorter recovery periods, reduced revision rates, and enhanced overall surgical success.
